# Tenecteplase versus alteplase before stroke thrombectomy: outcomes after system-wide transitions in Pennsylvania

**DOI:** 10.1007/s00415-024-12530-x

**Published:** 2024-07-03

**Authors:** Philipp Hendrix, Bradley A. Gross, Sepideh Allahdadian, Georgios S. Sioutas, Prateeka Koul, Antonio Corral Tarbay, Michael J. Lang, Visish M. Srinivasan, Alhamza R. Al-Bayati, Jiang Li, Anthony Noto, Raul G. Nogueira, Jan-Karl Burkhardt, Ramin Zand, Clemens M. Schirmer

**Affiliations:** 1 Department of Neurosurgery, Geisinger, Danville, PA USA; 2 Department of Neurosurgery, University of Pittsburgh Medical Center, Pittsburgh, PA USA; 3 Department of Neurology, Penn State Neuroscience Institute, Hershey, PA USA; 4 Department of Neurosurgery, Hospital of the University of Pennsylvania, Philadelphia, PA USA; 5 Department of Molecular and Functional Genomics, Weis Center for Research, Geisinger, Danville, PA USA; 6 Department of Neurology, Geisinger, Danville, PA USA; 7 Department of Neurology, University of Pittsburgh Medical Center, Pittsburgh, PA USA

**Keywords:** Ischemic stroke, Tenecteplase, Tissue-type plasminogen activator, Thrombectomy, Reperfusion

## Abstract

**Introduction:**

United States stroke systems are increasingly transitioning from alteplase (TPA) to tenecteplase (TNK). Real-world data on the safety and effectiveness of replacing TPA with TNK before large vessel occlusion (LVO) stroke endovascular treatment (EVT) are lacking.

**Methods:**

Four Pennsylvania stroke systems transitioned from TPA to TNK during the study period 01/2020–06/2023. LVO stroke patients who received intravenous thrombolysis with TPA or TNK before EVT were reviewed. Multivariate logistic analysis was conducted adjusting for age, sex, National Institute of Health Stroke Scale (NIHSS), occlusion site, last-known-well-to-intravenous thrombolysis time, interhospital-transfer and stroke system.

**Results:**

Of 635 patients, 309 (48.7%) received TNK and 326 (51.3%) TPA prior to EVT. The site of occlusion was the M1 middle cerebral artery (MCA) (47.7%), M2 MCA (25.4%), internal carotid artery (14.0%), tandem carotid with M1 or M2 MCA (9.8%) and basilar artery (3.1%). A favorable functional outcome (90-day mRS ≤ 2) was observed in 47.6% of TNK and 49.7% of TPA patients (*p* = 0.132). TNK versus TPA groups had similar rates of early recanalization (11.9% vs. 8.4%, *p* = 0.259), successful endovascular reperfusion (93.5% vs. 89.3%, *p* = 0.627), symptomatic intracranial hemorrhage (3.2% vs. 3.4%, *p* = 0.218) and 90-day all-cause mortality (23.1% vs. 21.5%, *p* = 0.491).

**Conclusions:**

This U.S. multicenter real-world clinical experience demonstrated that switching from TPA to TNK before EVT for LVO stroke resulted in similar endovascular reperfusion, safety, and functional outcomes.

**Supplementary Information:**

The online version contains supplementary material available at 10.1007/s00415-024-12530-x.

## Introduction

Randomized controlled trial data established intravenous tenecteplase (TNK) non-inferior to intravenous alteplase (TPA) in acute ischemic stroke within 4.5 h of symptom-onset [1–3]. Real-world clinical experience demonstrated that replacing TPA with TNK in acute ischemic stroke treatment appears safe and effective [4–7]. Many stroke systems in the United States have switched or are considering switching from TPA to TNK based on the recent trial data, guideline recommendations as well as the pharmacodynamic and pharmacokinetic advantages of TNK over TPA. Notably, TNK features a single bolus injection, which results in practical clinical advantages and improved workflow times [7–9]. The European Stroke Organisation (ESO) has issued a strong recommendation favoring TNK over TPA for thrombolysis-eligible patients with large vessel occlusion (LVO) stroke within 4.5 h of stroke-onset (moderate quality of evidence) [10]. The American Heart Association/American Stroke Association (AHA/ASA) guidelines suggest that the use of TNK over TPA may be reasonable (weak recommendation, moderate quality of evidence) [11]. Despite the recent thrombolytic landscape change, U.S. multicenter real-world data from patients receiving TNK prior to emergent endovascular treatment for LVO stroke is lacking. Here, we assessed the safety and effectiveness of switching to TNK before endovascular treatment of LVO stroke.

## Methods

**Fig. 1:**
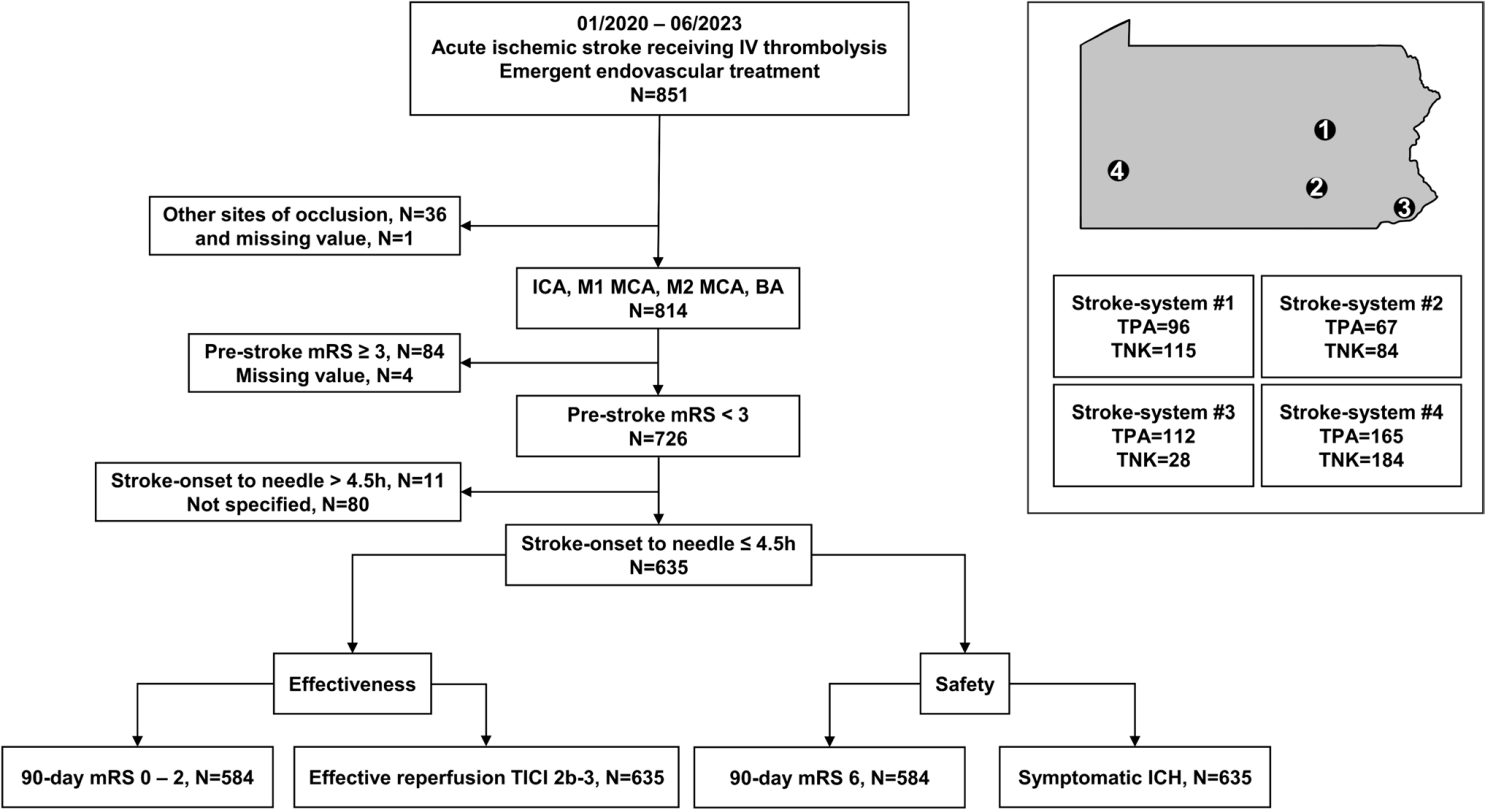
Study flowchart

We retrospectively reviewed consecutive acute ischemic stroke patients > 18 years who received intravenous thrombolysis (IVT) and underwent emergent endovascular treatment (EVT) between 01/2020 and 06/2023. Patients either received TPA (0.9 mg/kg) or TNK (0.25 mg/kg) and were then referred for EVT. Patients who did not primarily present to a comprehensive stroke center (CSC) required interhospital-transfer within the corresponding stroke system. Administration of IVT and eligibility for EVT followed contemporary AHA/ASA guidelines and stroke system protocols. During the study period, all stroke systems transitioned from TPA to TNK as their primary thrombolytic agent. The four stroke systems serve rural and urban populations across Pennsylvania and its neighboring states (Fig. 1). We prespecified the cohort of interest with the following criteria (i) LKW-to-IVT ≤ 4.5 h, (ii) intracranial internal carotid artery (ICA), M1 and M2 middle cerebral artery (MCA) with and without carotid tandem, and basilar artery (BA) occlusion, (iii) pre-stroke modified Rankin Scale (mRS) < 3 (Fig. 1). The primary effectiveness outcomes were 90-day mRS 0–2 and successful endovascular reperfusion (TICI 2b-3). The primary safety outcomes were 90-day all-cause mortality and symptomatic intracranial hemorrhage (sICH). Multivariate logistic regression analysis was conducted with prespecified adjustments for age, sex, National Institute of Health Stroke Scale (NIHSS), occlusion site, last-known-well (LKW) to intravenous thrombolysis time (IVT), interhospital-transfer, and stroke system (Data Supplement).

Pre-thrombectomy data points were available from three sites and were used to quantify early recanalization rates in a separate subset analysis. Early recanalization was defined as neurological improvement averting mechanical thrombectomy or evidence of recanalization of the target vessel with reperfusion > 50% on first target vessel angiography.

Institutional review board approval was obtained at each site. Consent was waived due to the retrospective nature of the study.

## Results

851 patients received IVT and EVT during the study era. Of 851 patients, 635 patients met the prespecified inclusion criteria. Among the 635 analyzed patients, 309 (48.7%) received TNK and 326 (51.3%) TPA prior to EVT (Fig. 1). Baseline demographics were comparable between TNK and TPA patients (Table 1), except for more patients in the TPA group requiring interhospital-transfer for EVT (*p* < 0.001). Univariate and multivariate analyses demonstrated similar pre-defined primary outcomes with TNK and TPA. A favorable functional outcome was observed in 47.6% of TNK and 49.7% of TPA patients (adjusted odds ratio (aOR) 0.72 (0.47–1.10), *p* = 0.13). TNK versus TPA groups had similar rates of successful endovascular reperfusion (93.5% vs. 89.3%, aOR of 1.19 (0.58–2.43), *p* = 0.627), symptomatic intracranial hemorrhage (3.2% vs. 3.4%, aOR of 0.54 (0.20–1.44, *p* = 0.218)) and 90-day all-cause mortality (23.1% vs. 21.5%, aOR of 1.21 (0.71–2.06, *p* = 0.491)).

**Table 1:** Demographics, procedure, and outcome metrics

	Tenecteplase (n = 309)	Alteplase (n = 326)	*P* value	Adjusted odds ratio (95% CI)	*P* value
*Baseline*					
Age median (IQR)	70 (61–78)	69 (58–79)	0.547		
Female	157/309 (50.8%)	158/326 (48.5%)	0.555		
Baseline NIHSS median (IQR)	17 (11–22)	17 (12–22)	0.985		
Pre-stroke mRS 0–1	284/309 (91.9%)	289/326 (88.7%)	0.167		
Laterality left	161/309 (52.1%)	154/326 (50.3%)	0.883		
Site of occlusion					
Internal carotid artery	41/309 (13.3%)	48/326 (14.7%)	0.787		
Middle cerebral artery M1	151/309 (48.9%)	152/326 (46.6%)			
Middle cerebral artery M2	81/309 (26.2%)	80/326 (24.5%)			
Basilar artery	10/309 (3.2%)	10/326 (3.1%)			
Tandem occlusion ICA and MCA	26/309 (8.4%)	36/326 (11.0%)			
CT-ASPECTS median (IQR)	10 (9–10)	9 (8–10)	0.104		
Primary presentation to CSC	147/308 (47.7%)	79/254 (31.1%)	< 0.001		
LKW to IV thrombolytic [mins]	123 (88–164)	120 (95–165)	0.566		
LKW to groin [mins]	198 (154–259)	227 (179–285)	< 0.001		
LKW to revascularization [mins]	238 (189–305)	260 (210–332)	< 0.001		
Groin to revascularization [mins]	29 (18–50)	31 (18–53)	0.403		
*Outcome*					
Successful reperfusion	289/309 (93.5%)	291/326 (89.3%)	0.056	1.19 (0.58–2.43)	0.627
24 h NIHSS median (IQR)	7 (2–16)	8 (3–15)	0.665	1.16 (0.285–4.75)	0.832
Intracranial hemorrhage					
Symptomatic ICH	10/309 (3.2%)	11/326 (3.4%)	0.923	0.54 (0.20–1.44)	0.218
Any parenchymal hematoma	41/309 (13.3%)	43/326 (13.2%)	0.977	0.84 (0.49–1.48)	0.561
Functional outcome at 90 days					
mRS 0–2 (favorable)	136/286 (47.6%)	148/298 (49.7%)	0.610	0.72 (0.47–1.10)	0.132
mRS 6 (death)	66/286 (23.1%)	64/298 (21.5%)	0.642	1.21 (0.71–2.06)	0.491

In a subset analysis of three centers with 517/851 patients, early recanalization was observed in 30/234 (12.8%) TNK and 25/283 (8.8%) TPA patients (*p* = 0.143). Following the prespecified inclusion criteria, early recanalization was observed in 20/168 (11.9%) TNK and 17/203 (8.4%) TPA cases (*p* = 0.259). Only lower baseline NIHSS was associated with early recanalization (*p* < 0.001).

## Discussion

This U.S. multicenter real-world clinical experience demonstrated that switching from TPA to TNK before EVT for LVO stroke resulted in similar endovascular reperfusion, safety, and functional outcomes.

In 2018, the tenecteplase versus alteplase before endovascular therapy for ischemic stroke (EXTEND-IA TNK) trial reported that TNK compared to TPA before LVO stroke was associated with a better early recanalization rate of the index vessel on first angiogram but similar final cerebral reperfusion rates [12]. Observational study data reported conflicting early recanalization rates of TNK and TPA, which could be explained with specifics to site of occlusion, needle-to-groin times, as well as thrombus properties [13–16]. Recently, a prespecified secondary analysis of LVO patients from the intravenous tenecteplase compared with alteplase for acute ischemic stroke in Canada (AcT) randomized controlled trial (RCT) was reported. The authors found similar reperfusion, safety, early reperfusion and functional outcomes with TNK and TPA in patients with LVO stroke [17]. Our prespecified inclusion criteria resemble those from the recent secondary analysis of LVO stroke patients from the AcT trial and our U.S. multicenter observational study findings parallel their observations. While meta-analyses and large scale studies support the overall safety of using TNK in acute ischemic stroke [18–20], our study solidifies the understanding of the subgroup of LVO stroke in the real-world setting and supports switching to TNK due to its safety, effectiveness and clinical advantages. However, the deciding to switch thrombolytics also requires considering factors such as transition logistics, costs and regulatory approvals. The current global shortage of thrombolytics may further complicate these processes [24].

Our study is limited by its retrospective, non-blinded and non-randomized character. Despite being one of the largest U.S. multicenter LVO stroke thrombectomy studies to date, the limited sample size may affect the comparison of low incidence variables such as sICH [20]. Optimal workflows among patients with rapid neurological improvement remain to be determined. Our study may have overlooked cases with early recanalization because they were potentially not referred for possible thrombectomy due to rapid neurological improvement—primarily, patients with low stroke burden. Similar to others, we found that achieving early recanalization was associated with a lower baseline NIHSS. The herein observed composite early recanalization rates (TNK 11.9% vs. TPA 8.4%) were similar to those from the AcT trial (TNK 9.2% vs. TPA 10.5%). However, ER rates are not yet routinely assessed in registries [20] and variations in institutional protocols complicate the retrospective assessment. We did not assess rates of extracranial hemorrhaghic complications or other adverse events such as angioedema [21, 22]. We did not assess distinct baseline demographic variables and despite adjusting for many established covariates including each stroke system, we could not evaluate the relevance of distinct risk criteria. Hyperglycemic patients are predisposed to TPA-associated sICH. It remains uncertain if comparable risks pertain to TNK [8, 23].

## Conclusion

In summary, this U.S. multicenter real-world clinical experience demonstrated that switching from TPA to TNK before EVT for LVO stroke resulted in similar endovascular reperfusion, safety, and functional outcomes. Therefore, switching to TNK is considered safe and effective.

### Supplementary Information

Below is the link to the electronic supplementary material.

Supplementary file1 (DOCX 345 kb)

## Data Availability

Data supporting the findings of this study are available upon reasonable request from the corresponding author when in agreement with all study site investigators.
