# Supplementary material for: Tenecteplase versus alteplase before stroke thrombectomy: outcomes after system-wide transitions in Pennsylvania
Source: J Neurol. 2024 Jul 3;271(8):5637–41. doi: 10.1007/s00415-024-12530-x (PMC11319427; doi:10.1007/s00415-024-12530-x)
Supplement: Supplementary file 1 — Supplementary file1 (DOCX 345 kb) [file 415_2024_12530_MOESM1_ESM.docx]

**SUPPLEMENT**

**Tenecteplase versus Alteplase before Stroke Thrombectomy: Outcomes after System-Wide Transitions in Pennsylvania**

**Statistical analysis**

Continuous variables were displayed as median with interquartile range. Qualitative variables were displayed as frequency with percent. Standard descriptive analysis was used to compare tenceteplase and alteplase groups. For univariate comparisons, chi-square, Fisher’s exact and Mann-Whitney-U tests were applied, as appropriate. Univariate and multivariate binary logistic regression was performed to obtain odds ratio and adjusted odds ratio including 95% confidence intervals. Adjustment was performed for pre-specified clinically relevant criteria. Inclusion criteria followed previously randomized controlled trial criteria to facilitate outcome comparison.[1] Data was missing at random. Cases with missing data points were discarded in the multivariate analysis models. Adjusted odds ratio were calculated for n=553 patients without missing variables (see Table below). Among these n=553 patients, in 41 patients the 90-day functional outcome metrics were missing. Hence, adjusted odds ratios for 90-day mRS 0-2 and mRS 6 were calculated in n=512 patients. Baseline metrics of cohorts with discarded cases (see Tables below) differed from the full n=635 cohort in the rate of patients with pre-stroke mRS 0-1 (TNK 92.2% vs TPA 87.0%, p=0.044). Further refining the multivariate analysis and adding pre-stroke mRS as additional covariate to the pre-specified covariates resulted in similar findings for all outcome metrics.

Supplement Table 1. Missing data percentages

|  | Missing n (%) |  |
| --- | --- | --- |
| ***Baseline (n=635)*** | | |
| Age median (IQR) | - |  |
| Female | - |  |
| Baseline NIHSS median (IQR) | 10 (1.6%) |  |
| Pre-stroke mRS 0-1 | - |  |
| Laterality left | - |  |
| Site of occlusion | - |  |
| Internal carotid artery |  |  |
| Middle cerebral artery M1 |  |  |
| Middle cerebral artery M2 |  |  |
| Basilar artery |  |  |
| Tandem occlusion ICA & MCA |  |  |
| CT-ASPECTS median (IQR) | 109 (17.1%) |  |
| Primary presentation to CSC | 73 (11.5%) |  |
| LKW to IV thrombolytic [mins] | - |  |
| LKW to groin [mins] | 4 (0.6%) |  |
| LKW to revascularization [mins]* | 12 (1.9%) |  |
| Groin to revascularization [mins]* | 12 (1.9%) |  |
| ***Outcome*** | | |
| Successful reperfusion | - |  |
| 24h NIHSS median (IQR) | 39 (6.1%) |  |
| Intracranial hemorrhage | - |  |
| Symptomatic ICH |  |  |
| Any parenchymal hematoma |  |  |
| Functional outcome at 90 d | 51 (8.0%) |  |
| mRS 0 – 2 (favorable) |  |  |
| mRS 6 (death) |  |  |

* End point not reached due to index vessel recanalization in 10 TNK and 7 TPA cases.

Supplement Table 2. Adjusted analysis cohort after discarding cases with missing covariate variables.

|  | Tenecteplase  (n=307) | Alteplase  (n=246) | P-value | Missing  n (%) |
| --- | --- | --- | --- | --- |
| ***Baseline (n=553)*** |  |  |  |  |
| Age median (IQR) | 70 (61 – 78) | 69 (58 – 79) | 0.596 | - |
| Female | 157 (51.1%) | 119 (48.4%) | 0.518 | - |
| Baseline NIHSS median (IQR) | 17 (11 – 22) | 17 (12 – 21) | 0.861 | - |
| Pre-stroke mRS 0-1 | 283 (92.2%) | 214 (87.0%) | 0.044 | - |
| Laterality left | 159 (51.8%) | 125 (50.8%) | 0.630 | - |
| Site of occlusion |  |  |  | - |
| Internal carotid artery | 41 (13.4%) | 37 (15.0%) | 0.715 |  |
| Middle cerebral artery M1 | 149 (48.5%) | 116 (47.2%) |  |  |
| Middle cerebral artery M2 | 81 (26.4%) | 61 (24.8%) |  |  |
| Basilar artery | 10 (3.3%) | 5 (2.0%) |  |  |
| Tandem occlusion ICA & MCA | 26 (8.5%) | 27 (11.0%) |  |  |
| CT-ASPECTS median (IQR) | 10 (9 – 10) | 9 (8 – 10) | 0.069 | 37 (6.7%) |
| Primary presentation to CSC | 146 (47.6%) | 77 (31.3%) | <0.001 | - |
| LKW to IV thrombolytic [mins] | 122 (88 – 165) | 119 (92 – 159) | 0.899 | - |
| LKW to groin [mins] | 198 (153 – 259) | 225 (181 – 284) | <0.001 | 4 (0.7%) |
| LKW to revascularization [mins]* | 238 (189 – 305) | 264 (213 – 333) | <0.001 | 9 (1.6%) |
| Groin to revascularization [mins]* | 29 (18 – 50) | 33 (18 – 54) | 0.220 | 9 (1.6%) |
| ***Outcome*** |  |  |  |  |
| Successful reperfusion | 287 (93.5%) | 224 (91.1%) | 0.284 | - |
| 24h NIHSS median (IQR) | 7 (2 – 16) | 8 (3 – 16) | 0.609 | 38 (6.8%) |
| Intracranial hemorrhage |  |  |  | - |
| Symptomatic ICH | 10 (3.3%) | 11 (4.5%) | 0.458 |  |
| Any parenchymal hematoma | 41 (13.4%) | 31 (12.6%) | 0.794 |  |
| Functional outcome at 90 d |  |  |  | 41 (7.4%) |
| mRS 0 – 2 (favorable) | 135 (47.4%) | 114 (50.2%) | 0.521 |  |
| mRS 6 (death) | 66 (23.2%) | 45 (19.8%) | 0.363 |  |

* End point not reached due to index vessel recanalization in 10 TNK and 7 TPA cases.

**Outcome variable definition**

Cerebral imaging (CT head or magnetic resonance imaging) 24-36 hours after thrombolysis was assessed to evaluate for intracranial hemorrhage. Any deterioration in National Institutes of Health stroke scale score of 4 attributed to parenchymal hemorrhage type 2 was considered a symptomatic ICH as per Safe Implementation of Thrombolysis in Stroke-Monitoring Study (SITS-MOST) criteria.[2] Any parenchymal hematoma was defined as a composite of parenchymal hematoma type 1 and type 2 irrespective of neurological deterioration.

**Supplement Figure 1.** Functional outcomes of TNK and TPA cohorts.


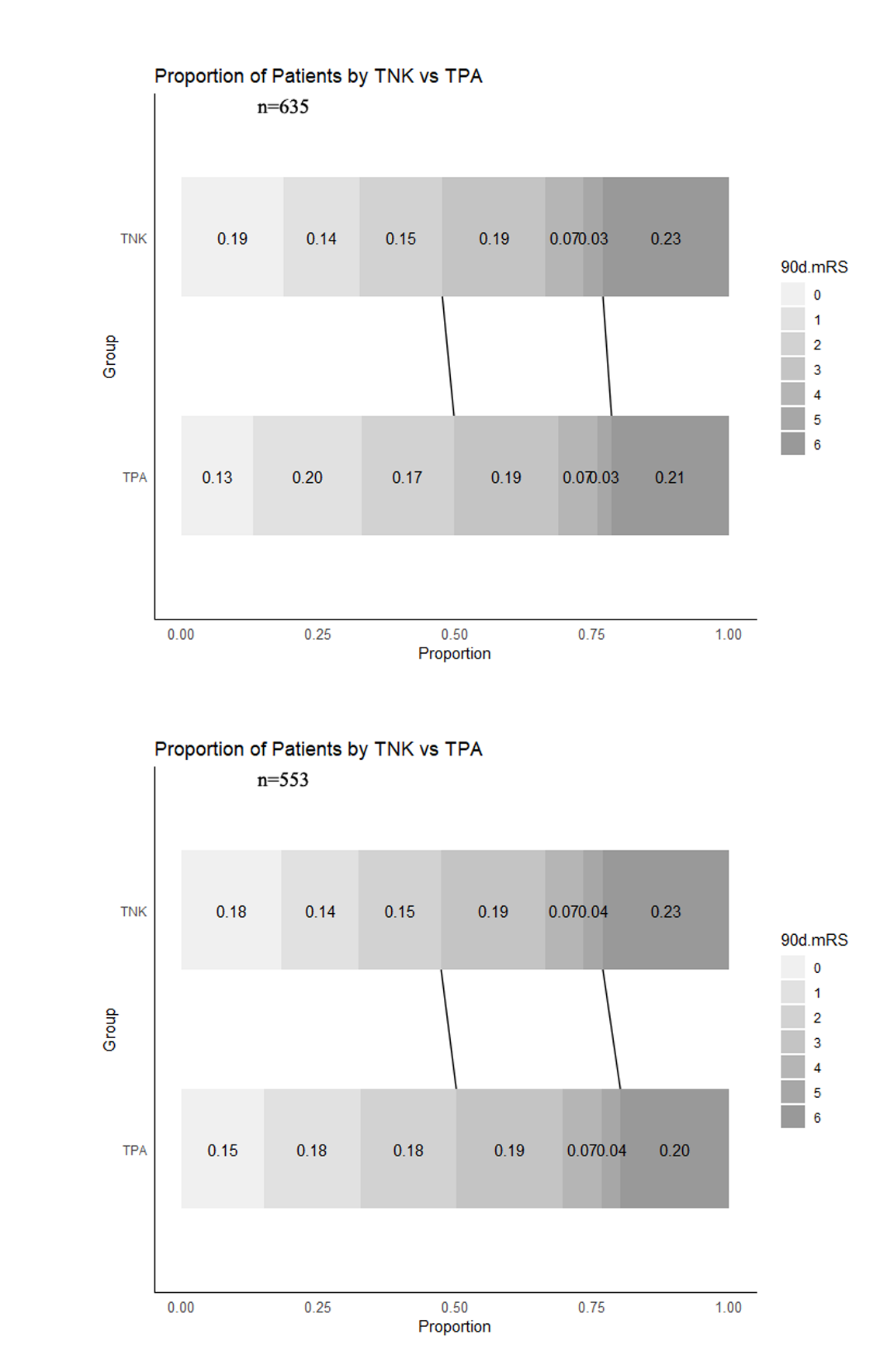


**References (Supplement)**

1. Bala F, Singh N, Buck B, et al (2023) Safety and Efficacy of Tenecteplase Compared With Alteplase in Patients With Large Vessel Occlusion Stroke: A Prespecified Secondary Analysis of the ACT Randomized Clinical Trial. JAMA Neurol 80:824–832. https://doi.org/10.1001/jamaneurol.2023.2094

2. Mazya M, Egido JA, Ford GA, et al (2012) Predicting the Risk of Symptomatic Intracerebral Hemorrhage in Ischemic Stroke Treated With Intravenous Alteplase: Safe Implementation of Treatments in Stroke (SITS) Symptomatic Intracerebral Hemorrhage Risk Score. Stroke 43:1524–1531. https://doi.org/10.1161/STROKEAHA.111.644815
